# Comparative efficacy of experimental inactivated and live-attenuated chimeric porcine circovirus (PCV) 1-2b vaccines derived from PCV1 and PCV2b isolates originated in China

**DOI:** 10.1186/s12985-015-0338-9

**Published:** 2015-07-30

**Authors:** Jizong Li, Tianqi Yu, Xiaobo Wang, Jinzhu Zhou, Ruxia Gao, Feipeng Zhang, Xing Gao, Song Gao, Xiufan Liu

**Affiliations:** Animal Infectious Disease Laboratory, Ministry of Agriculture, Yangzhou University, Yangzhou, Jiangsu 225009 People’s Republic of China; Jiangsu Co-Innovation Centre for Prevention and Control of Important Animal Infectious Diseases and Zoonoses, College of Veterinary Medicine, Yangzhou University, Yangzhou, Jiangsu 225009 People’s Republic of China

**Keywords:** Porcine circovirus type 2, Inactivated and live-attenuated chimeric PCV1-2b vaccine, Inactivated PCV2b vaccine, Efficacy

## Abstract

**Background:**

Porcine circovirus type-2b (PCV2b) is recognized as the etiological agent of the various clinical manifestations of porcine circovirus-associated disease (PCVAD). Previous studies have demonstrated effectiveness of chimeric PCV1-2 vaccines against PCV2b challenge. In this study, the efficacy of inactivated and live-attenuated (2 × 10^3.5^ or 2 × 10^4.0^ 50 % tissue culture infective dose [TCID_50_] dose) chimeric PCV1-2b vaccines was compared side-by-side in conventional pigs.

**Methods:**

Twenty-seven non-PCV2 viremic pigs without PCV2-specific antibody were randomly divided into six groups, including four vaccinated and challenged groups, a nonvaccinated challenged group, and a mock group. All pigs except those in the mock group were challenged at 28 days post vaccination (DPV) using PCV2b.

**Results:**

Both inactivated and live-attenuated chimeric PCV1-2b vaccines induced a robust antibody responses, and significantly decreased microscopic lesion and lower viral loads in serum or superficial inguinal lymph nodes (SILN) compared with that in the nonvaccinated challenged group. PCV2 antibody titers decreased after 7 days post challenge (DPC) in pigs administered the inactivated PCV1-2b vaccine and they were lower than those in pigs inoculated with live-attenuated PCV1-2b on the day of necropsy. Moreover, no viremia was present in pigs inoculated with live-attenuated PCV1-2b vaccine at 21 DPC regardless of the dose difference.

**Conclusions:**

The results demonstrated that both inactivated and live-attenuated chimeric PCV1-2b vaccines were effective to induce protective immunity against PCV2b infection.

## Background

Porcine circovirus type 2 (PCV2) is the primary causative agent of the postweaning multisystemic wasting syndrome (PMWS). This disease was first identified and reported in western Canada in 1991 [1, 2]. Along with PMWS, PCV2 is also associated with a number of diseases and syndromes collectively called porcine circovirus-associated disease (PCVAD) [3–5]. PMWS is recognized as one of the most economically important diseases in the global swine industry.

Porcine circovirus (PCV) is a small, nonenveloped virus with a single-strand circular DNA genome of 1.7 kb in the family *Circoviridae* [6]. Two genotypes of PCV have been identified, PCV type 1 (PCV1) and PCV type 2 (PCV2). In general, it is known that PCV1 is nonpathogenic, however, PCV1 can produce pathology in the lungs of porcine fetuses [7]. Whereas the virus isolated from pig with PMWS has been designated PCV2 [1, 8]. PCV2 genome has three major open reading frames (ORFs). ORF1 encodes viral replication-associated proteins [9, 10], ORF2 encodes the viral immunogenic protein, which has been the target for developing the recombinant vaccines [6, 11], ORF3 encodes an apoptosis associated protein that plays important roles in the pathogenesis of PCV2 [12]. Currently, PCV2 can be further divided into three main subtypes: PCV2a, which is further subdivided into five clusters, 2A to 2E; PCV2b, which is subdivided into three clusters, 1A to 1C [13, 14]; and PCV2c, which has only been reported in Denmark [15]. PCV2a and PCV2b have both been associated with clinical PCVAD of varying degrees of severity [16, 17]. PCV2b has been suggested as being potentially more pathogenic than PCV2a [18, 19].

It has been shown that vaccinations are a major tool for reducing PCVAD losses in swine populations. There are currently several types of commercial vaccine products available worldwide and they differ in antigen. One vaccine is based on the inactivated PCV2a virus [6]. Two subunit vaccines are based on capsid protein expressed in the baculovirus system. The other chimeric PCV1-2a vaccine contains the genomic backbone of PCV1 with the capsid gene replaced by that of PCV2a [20, 21]. Several published articles revealed that these inactivated vaccines were effective in inducing neutralizing antibodies and in reducing PCV2 viremia [22–24]. Moreover, it has been reported that pigs vaccinated with 10^3.5^ or 10^4.0^ 50 % tissue culture infective dose (TCID_50_)/ml dose live-attenuated chimeric PCV1-2 vaccine developed high levels of antibody and the vaccinated pigs were fully protected against challenge with PCV2 [24–27]. However, there has been less side-by-side comparison between the efficacy of experimental inactivated and live-attenuated PCV1-2b vaccines and the live-attenuated vaccines with different doses (2 × 10^3.5^ or 2 × 10^4.0^ TCID_50_ dose) in growing pigs. In this study we demonstrated that pigs can be effectively protected against PCV2b challenge by vaccination with inactivated or live-attenuated PCV1-2b vaccines.

## Results

### Clinical presentation

Clinical signs and pathological lesions were not found in the vaccinated and mock groups, whereas two pigs in the challenged group were markedly depressed and had fever symptoms for 1 day (40.2 °C-41.4 °C). One pig in the inactivated PCV2b vaccine group died after routine blood collection on 7 days post vaccination (DPV) because of massive hemorrhages in the neck area. The average daily weight gain (ADWG) ranged from 0.30 to 0.40 kg/day during the growing period (3–7 weeks of age), with no significant differences among groups. The ADWG ranged from 0.51 to 0.69 kg/DPC (7–10 weeks of age), with no significant differences among groups (Table 2).

**Table 1:** Grouping and treatment of experimental pigs

Group	Vaccine	Vaccine dose (TCID_50_)	Vaccination day	Challenge dose (TCID_50_)	Challenge day
1	Live-attenuated PCV1-2b	2 × 10^3.5^	21	2 × 10^4.8^	49
2	Live-attenuated PCV1-2b	2 × 10^4.0^	21	2 × 10^4.8^	49
3	Inactivated PCV1-2b vaccine	2 × 10^5.0^	21	2 × 10^4.8^	49
4^a^	Inactivated PCV2b vaccine X	2 ml	21	2 × 10^4.8^	49
5	Challenge without vaccination	-	-	2 × 10^4.8^	49
6	Mock group	-	-	-	-

^a^One pig died after routine bleeding at 7 DPV due to massive hemorrhaging

**Table 2:** Fever and ADWG from challenge day (28 DPV) to necropsy day (21 DPC)

Group	Days with fever (≥40 °C)	Body weight			ADWG before challenge	ADWG after challenge
		Day at vaccination	Day at challenge	28 DPC		
1	0	9.54 ± 0.34	20.84 ± 0.63	35.38 ± 1.06	0.40 ± 0.01	0.69 ± 0.03
2	0	9.58 ± 0.81	20.02 ± 1.79	32.16 ± 4.03	0.37 ± 0.04	0.58 ± 0.11
3	0	6.58 ± 0.26	16.48 ± 0.95	27.18 ± 1.94	0.35 ± 0.03	0.51 ± 0.05
4	0	6.85 ± 0.38	18.15 ± 1.21	30.30 ± 1.87	0.40 ± 0.03	0.58 ± 0.03
5	0.40 ± 0.25	7.10 ± 0.50	15.40 ± 1.12	26.88 ± 2.08	0.30 ± 0.04	0.55 ± 0.05
6	0	6.55 ± 0.25	16.15 ± 1.35	31.10 ± 2.80	0.34 ± 0.04	0.61 ± 0.07

*ADWG* average daily weight gain, *DPC* days post challenge, *DPV* days post vaccination

### Anti-PCV2 IgG antibodies

**Fig. 1:**
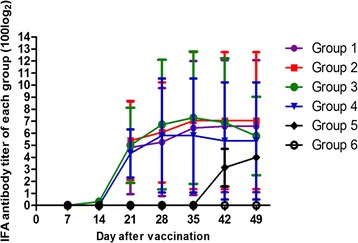
Dynamics of mean values of IFA antibody titers in each group. The grouping and treatment of the experimental pigs are summarized in Table 1

No PCV2-specific antibodies were detected in all five groups at the time of vaccination, and PCV2-specific antibodies were not detected in the mock group throughout the experiment. Seroconversion to PCV2-specific antibodies was first detected at 14 DPV in vaccinated pigs (the IFA antibody titers ranged from 1:10 to 1:400), and all five pigs in the vaccinated groups were seropositive against PCV2 by 21 DPV (the IFA antibody titers ranged from 1:400 to 1:6400). Pigs vaccinated with the inactivated or 2 × 10^4.0^ TCID_50_ dose live-attenuated vaccine had higher and earlier PCV2 antibodies. On 14 DPV, 80 % (4 out of 5) of the pigs in the inactivated and 2 × 10^4.0^ TCID_50_ dose live-attenuated vaccine groups were seropositive, the IFA antibody titers were 0, 1:10, 1:10, 1:100, and 1:400 and 0, 1:10, 1:10, 1:10, and 1:400, respectively, whereas only 40 % (2 out of 5) of the pigs vaccinated with 2 × 10^3.5^ TCID_50_ dose live-attenuated vaccine had seroconversion to PCV2; the IFA antibody titers were 0, 0, 0, 1:10, and 1: 400. The IFA antibody titers peaked at 35 DPV or 42 DPV and remained high on the day of necropsy in the pigs vaccinated with the 2 × 10^4.0^ TCID_50_ dose and the 2 × 10^3.5^ TCID_50_ dose; however, PCV2 antibody titers decreased from 35 DPV in pigs vaccinated with inactivated PCV1-2b. Regarding commercial inactivated PCV2b vaccine X, the PCV2 antibody levels were highest at 28 DPV (the mean IFA antibody titer was 1:5600) and then declined rapidly at 35 DPV. For the nonvaccinated challenged group, all five pigs seroconverted to PCV2-specific antibodies at 14 DPC (the IFA antibody titers ranged from 1:400 to 1:1600). There were no significant differences in the mean PCV2-specific antibodies between any of the vaccinated groups (Fig. 1).

### Neutralizing antibodies

**Fig. 2:**
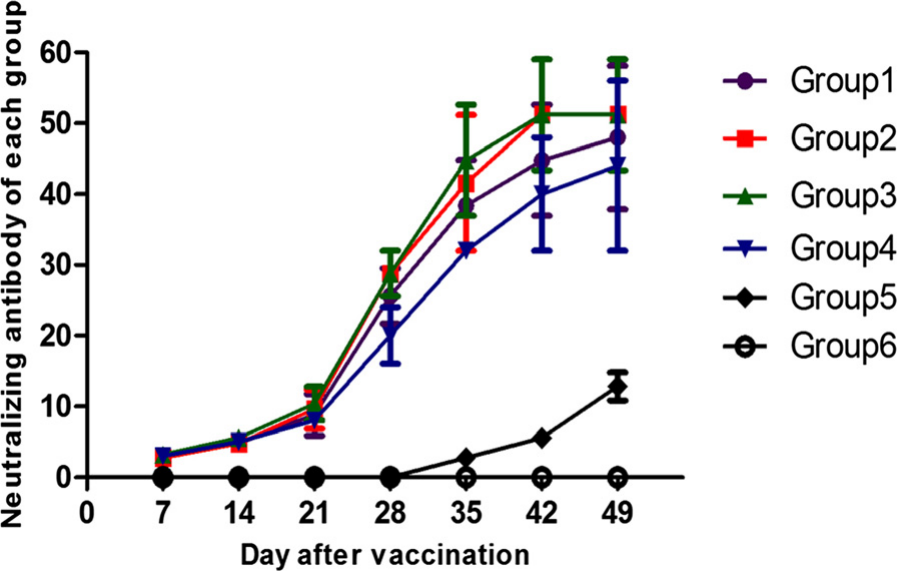
Mean values of the serum neutralizing antibody (NA) titers in the different groups. The grouping and treatment of the experimental pigs are summarized in Table 1

On the day of vaccination, no NA antibodies were detected among all five groups. Remarkable amounts of NA were detected in the vaccinated pigs at 14 DPV, and the NA titers in pigs vaccinated with inactivated PCV1-2b were higher than those in pigs vaccinated with the live-attenuated vaccine; the NA titers were 4.8 ± 0.80, 4.8 ± 0.80, 5.6 ± 0.98, and 5.0 ± 1.00 in groups 1, 2, 3, and 4, respectively. NA titers increased significantly on the day of challenge (28 DPV) when they were 25.60 ± 3.92, 28.80 ± 3.20, 28.80 ± 3.20, and 20.0 ± 4.00, respectively. In general, the inactivated and 2 × 10^4.0^ TCID_50_ dose live-attenuated vaccines induced a higher level of NA titers than 2 × 10^3.5^ TCID_50_ dose live-attenuated and commercial inactivated PCV2b vaccines during the course of the study, which corresponded to the PCV2 IFA antibody titers. The NA titers were not detected in pigs in the mock group throughout the experiment (Fig. 2).

### Detection of PCV2 DNA in serum

PCV2 DNA was not detected in any pigs before challenge or in pigs in the mock group at any sampling time point. The loads of PCV2 DNA amounts per milliliter of serum are summarized in Table 3.

**Table 3:** Prevalence and group mean log_10_ of PCV2 genomic copies/ml serum in pigs at different DPC

Group	7 DPC (mean log_10_ of PCV2 copies)	14 DPC (mean log_10_ of PCV2 copies)	21 DPC (mean log_10_ of PCV2 copies)
1	1/5 (1.56)	2/5 (3.01)	0/5 (0.00)
2	0/5 (0.00)	1/5 (1.33)	0/5 (0.00)
3	0/5 (0.00)	0/5 (0.00)	1/5 (1.11)
4	1/4 (1.74)	1/4 (1.61)	1/4 (1.42)
5	5/5 (7.39)	5/5 (8.22)	5/5 (7.37)
6	0/2 (0.00)	0/2 (0.00)	0/2 (0.00)

*DPC* days post challenge, *DPV* days post vaccination

In the 2 × 10^3.5^ TCID_50_ dose live-attenuated PCV1-2b vaccine group, PCV2 virus DNA was detected in one of five pigs with a load of 10^1.56^ PCV2 genomic copy numbers at 7 DPC. At 14 DPC, two of five pigs were found to have approximately 10^3.01^ PCV2 genomic copy numbers, and no PCV2 genomic copy numbers were detected in any of five pigs at 21 DPC.

In the 2 × 10^4.0^ TCID_50_ dose live-attenuated PCV1-2b vaccine group, no pig had PCV2 genomic copy numbers after challenge, except one pig that harbored 10^1.33^ PCV2 genomic copy numbers at 14 DPC.

In the inactivated PCV1-2b vaccine group, no PCV2 genomic copy numbers were detected in the pigs after challenge, except for one pig that had approximately 10^1.11^ PCV2 genomic copy numbers at 21 DPC.

For the commercial inactivated PCV2b vaccine control group, one out of four pigs had PCV2 genomic copy numbers that varied from 10^1.42^ to 10^1.74^ at 7 to 21 DPC.

**Fig. 3:**
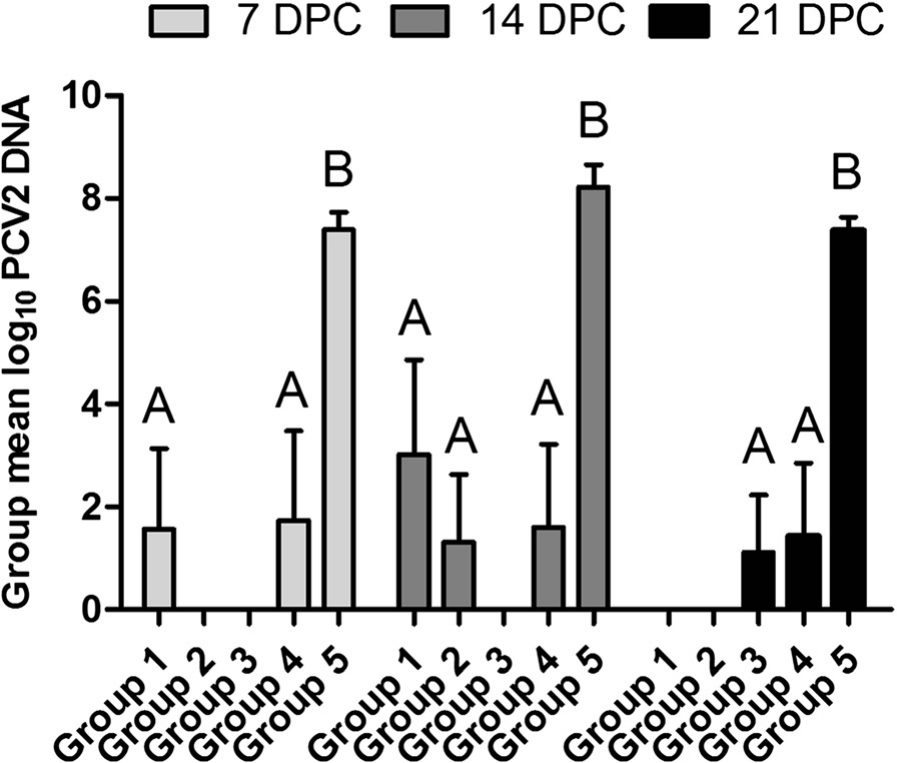
Comparison of the PCV2 DNA loads in sera of the differently treated pigs challenged with PCV2. The PCV2 genomic copy load was presented as a log_10_ value of PCV2 genomic copy load per 1 ml of sera. Different letters (A, B) indicate significant differences among different groups at 7, 14, or 21 DPC (*P* < 0.05)

Figure 3 shows a comparison of the PCV2 DNA load in sera of the pigs challenged with PCV2. The PCV2 DNA loads in serum were not significantly different among the vaccinated groups; however, the PCV2 DNA loads in nonvaccinated challenged pigs were significantly higher than those in the vaccinated groups (*P* < 0.05).

### Detection of PCV2 DNA in superficial inguinal lymph nodes

**Fig. 4:**
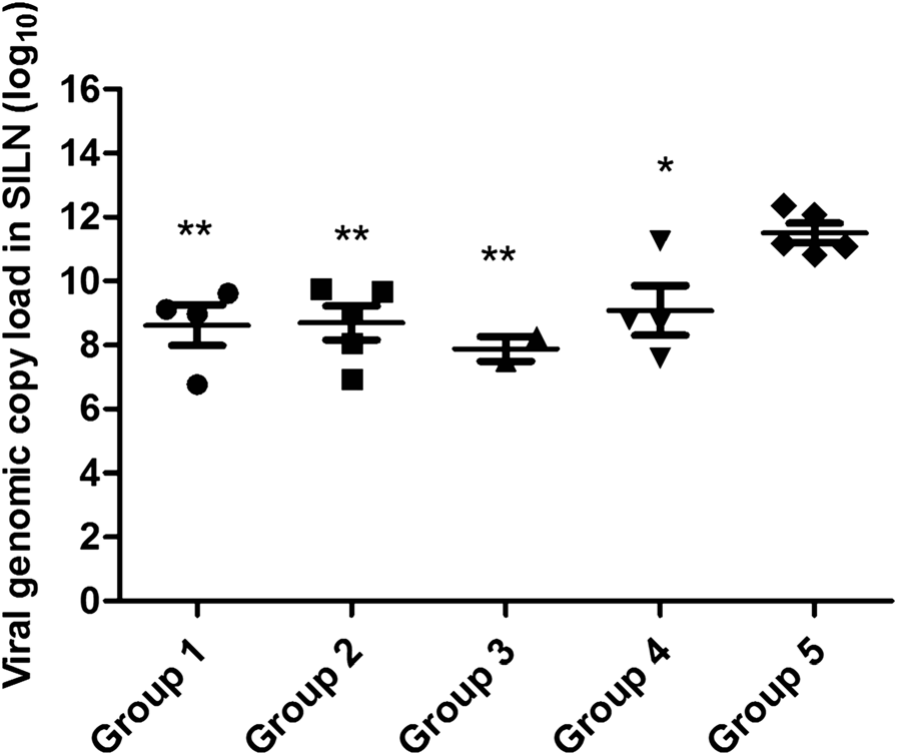
PCV2 DNA copies/g loads in SILN tissues collected on the day of necropsy. PCV2 DNA was extracted from 50 μg of homogenized SILN tissues and subjected to real-time PCR amplification. The PCV2 genomic copy loads are represented as a log_10_ value per 1 g of SILN tissue (y axis) (*P < 0.05; **P < 0.01)

At necropsy (DPC 21), PCV2 genomic DNA was detected in the SILN in four of five pigs in group 1, in two of five pigs in group 3, and in all pigs in groups 2, 4, and 5. The mean PCV2 viral genomic copies/g loads in homogenized SILN in samples that tested positive were 10^8.62^ in group 1, 10^8.70^ in group 2, 10^7.89^ in group 3, 10^9.08^ in group 4, and 10^11.50^ in group 5 (Fig. 4). The SILN viral loads in pigs inoculated with the inactivated PCV1-2b vaccine were slightly lower than those in pigs immunized with live-attenuated or commercial inactivated PCV2b vaccine, although the difference was not statistically significant. Median PCV2 DNA copy loads in vaccinated pigs were significantly lower than those in nonvaccinated challenged pigs, in groups 1 and 5 (*P* = 0.0032), in groups 2 and 5 (*P* = 0.0019), in groups 3 and 5 (*P* = 0.0012), and in groups 4 and 5 (*P* = 0.0154).

### Gross and microscopic lesions

**Fig. 5:**
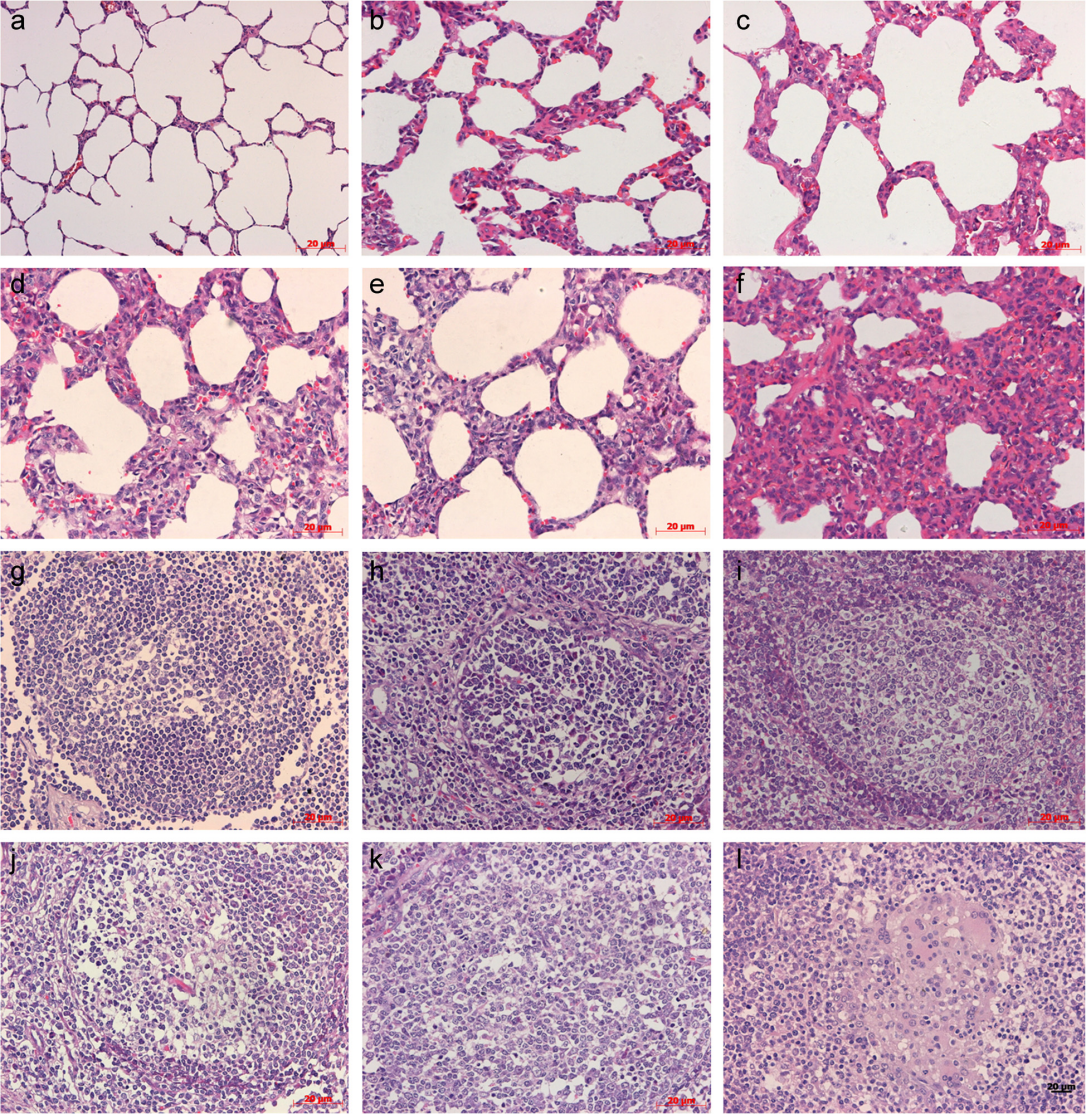
Histopathological lesions in experimental conventional pigs. **a** No markable microscopic lesions in the lung of mock group pigs. **b** Slight lymphoplasmacytic and histiocytic bronchointerstitial pneumonia in the lung of a pig vaccinated with 2×10^4.0^ TCID_50_ dose attenuated PCV1-2b. **c** Mild lymphoplasmacytic and histiocytic bronchointerstitial pneumonia in the lung of a pig vaccinated with inactivated PCV1-2b. **d** Moderate lymphoplasmacytic and histiocytic bronchointerstitial pneumonia in the lung of a pig vaccinated with 2×10^3.5^ TCID_50_ dose attenuated PCV1-2b. **e** Conspicuous lymphoplasmacytic and histiocytic bronchointerstitial pneumonia in the lung of a pig vaccinated with commercial inactivated PCV2b vaccine X. **f** Severe lymphoplasmacytic and histiocytic bronchointerstitial pneumonia in the lung of a nonvaccinated challenged pig. **g** No remarkable microscopic lesions in the lymph nodes of a pig in the mock group. **h** Slight lymphoid depletion (LD) in lymph node follicles of a pig vaccinated with 2×10^4.0^ TCID_50_ dose attenuated PCV1-2b. **i** Mild LD in lymph nodes follicles of a pig vaccinated with inactivated PCV1-2b. **j** Moderate LD in lymph nodes follicles of a pig vaccinated with 2×10^3.5^ TCID_50_ dose attenuated PCV1-2b. **k** Conspicuous LD in lymph nodes follicles of a pig vaccinated with commercial inactivated PCV2b vaccine X. **l** Moderate histiocytic replacement (HR) in lymph node follicles of a nonvaccinated challenged pig. Bar = 20 μm (400×)

No gross or microscopic lesions were observed in pigs in the mock group (Fig. 5a, g). At necropsy, the enlargement of lymph nodes were observed in the vaccinated pigs and generally ranged from mild to moderate; two pigs (two-times normal size) in the inactivated PCV1-2b vaccine group and one pig in 2 × 10^4.0^ TCID_50_ dose live-attenuated vaccine group had mild enlargement, whereas two pigs (three-times normal size) in the commercial inactivated PCV2b vaccine X group and one pig in 2 × 10^3.5^ TCID_50_ dose live-attenuated vaccine group had moderate swelling. The lymph nodes of all five pigs in the nonvaccinated challenged group were moderately to severely enlarged.

In vaccinated pigs, two pigs in the commercial inactivated PCV2b vaccine X group had lung lesions and only one pig in the inactivated or live-attenuated PCV1-2b vaccine group presented interstitial pneumonia; this was significant differences (one or two pigs versus four pigs) compared with nonvaccinated challenged pigs (Table 4, Fig. 5b-f).

**Table 4:** Distribution of histopathological lesions in different tissues from experimental pigs challenged with PCV2

Group	No. of pigs with lesions/no. examined											
	Lymph node		Tonsil		Spleen		Lung	Liver	Kidney	Heart	Thymus	Intestine
	LD	HR	LD	HR	LD	HR						
1	1/5	1/5	1/5	0/5	1/5	0/5	1/5	1/5	0/5	0/5	0/5	0/5
2	1/5	0/5	0/5	0/5	0/5	0/5	1/5	0/5	0/5	0/5	0/5	0/5
3	1/5	1/5	1/5	0/5	0/5	0/5	1/5	0/5	0/5	0/5	0/5	0/5
4	2/4	1/4	1/4	0/4	1/4	0/4	2/4	1/4	1/4	0/4	0/4	0/4
5	5/5	4/5	3/5	3/5	3/5	2/5	4/5	3/5	3/5	0/5	0/5	0/5
6	0/2	0/2	0/2	0/2	0/2	0/2	0/2	0/2	0/2	0/2	0/2	0/2

*HR* histiocytic replacement, *LD* lymphoid depletion

**Table 5:** Detection of PCV2 antigen in lymph node, tonsil, spleen, and thymus of experimental pigs at 21 DPC

Group	No. of pigs testing positive/no.tested			
	Lymph node	Tonsil	Spleen	Thymus
1	1/5	0/5	0/5	0/5
2	0/5	0/5	0/5	0/5
3	1/5	0/5	0/5	0/5
4	1/4	0/4	0/4	0/4
5	5/5	2/5	1/5	1/5
6	0/2	0/2	0/2	0/2

*DPC* days post challenge

Lymphoid depletion (LD) of lymph node follicles was detected in one pig in the inactivated or live-attenuated PCV1-2b vaccine groups and in two pigs in the commercial inactivated PCV2b vaccine X group. Histiocytic replacement (HR) of lymph node follicles was found in one pig in the inactivated or 2 × 10^3.5^ TCID_50_ dose live-attenuated vaccine group, commercial inactivated PCV2b vaccine X group, and in no pigs in the 2 × 10^4.0^ TCID_50_ dose live-attenuated vaccine group, compared with five pigs that displayed LD and four of five pigs that displayed HR in the nonvaccinated challenged group (Table 4, Fig. 5h-l).

LD of the tonsil follicles was observed in one pig in the inactivated or 2 × 10^3.5^ TCID_50_ dose live-attenuated vaccine group, commercial inactivated PCV2b vaccine X group, and in no pigs in the 2 × 10^4.0^ TCID_50_ dose live-attenuated PCV1-2b vaccine group, whereas LD or HR was detected in the tonsil follicles of three pigs in the nonvaccinated challenged group.

LD of the spleen follicles was found in one pig in the 2 × 10^3.5^ TCID_50_ dose live-attenuated PCV1-2b vaccine group, commercial inactivated PCV2b vaccine X group, and in no pigs in the inactivated or 2 × 10^4.0^ TCID_50_ dose live-attenuated vaccine group, but three of five pigs exhibited LD and two of five pigs exhibited HR effects.

In general, the characteristic LD and HR effects were lower in vaccinated pigs compared with the nonvaccinated challenged pigs. Results regarding the presence of lesions in all tissues and organs tested are summarized in Table 4.

### Amount of PCV2 antigen

**Fig. 6:**
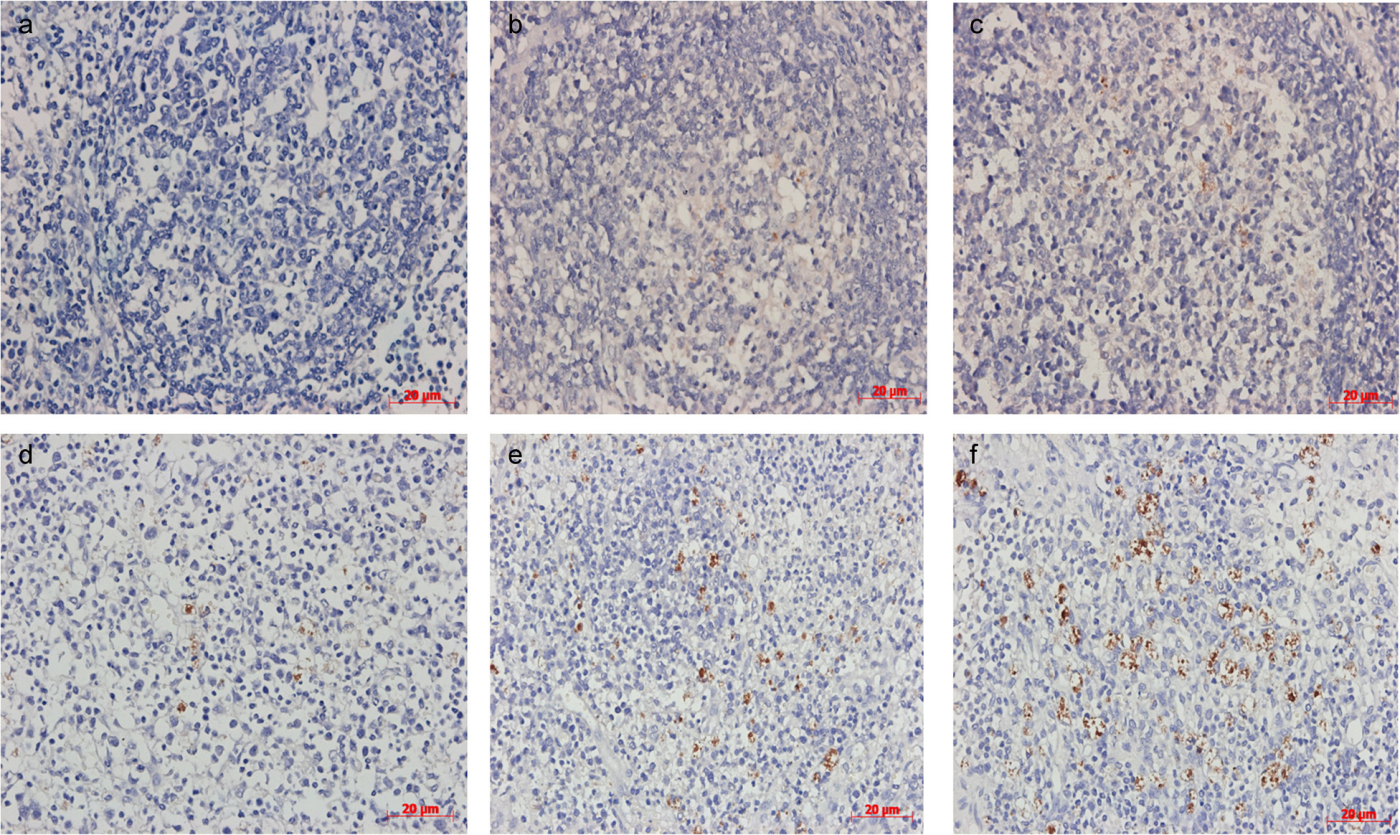
Immunohistochemical detection of PCV2 antigen in conventional pigs. **a** No detection of PCV2 antigen in lymph nodes of mock pigs. **b** Very small amounts of PCV2 antigen in lymph nodes of a pig vaccinated with 2×10^4.0^ TCID_50_ dose attenuated PCV1-2b. **c** Low amounts of PCV2 antigen in nuclei of histiocytes and occasional lymphocytes of a pig vaccinated with inactivated PCV1-2b. **d** Small amounts of PCV2 antigen in nuclei of histiocytes and occasional lymphocytes of a pig vaccinated with 2×10^3.5^ TCID_50_ dose attenuated PCV1-2b. **e** Moderate amounts of PCV2 antigen in nuclei of histiocytes and occasional lymphocytes of a pig vaccinated with commercial inactivated PCV2b vaccine X. **f** High amounts of PCV2 antigen in nuclei of histiocytes and occasional lymphocytes of a nonvaccinated challenged pig. Bar = 20 μm (400×). Hematoxylin-counterstained sections

At necropsy, the incidence and amount of PCV2 antigen were reduced in vaccinated pigs compared with nonvaccinated challenged group pigs. PCV2 antigen was not detected in the lymph node tissues of mock group (Table 5, Fig. 6a) and there were very small amounts of PCV2 antigen in pigs vaccinated with 2 × 10^4.0^ TCID_50_ dose live-attenuated PCV1-2b (Fig. 6b). Low amounts of PCV2 antigen were detected in one pig in the inactivated PCV1-2b vaccine group (Table 5 Fig. 6c) Small amounts were found in one pig in the 2 × 10^3.5^ TCID_50_ dose live-attenuated PCV1-2b vaccine group (Table 5, Fig. 6d). A moderate amount was found in one pig in the commercial inactivated PCV2b X vaccine group (Table 5, Fig. 6e). High amounts of PCV2 antigen were detected in the lymph nodes of all nonvaccinated challenged pigs (Table 5, Fig. 6f). No PCV2 antigen was detected in the tonsil, spleen, and thymus of any vaccinated pigs. However, varying amounts of PCV2 antigen were detectable in tonsils of two pigs and in spleen and thymus of one pigs in the nonvaccinated challenged group (Table 5).

## Discussion

Currently, all commercially available PCV2 vaccines are inactivated or subunit vaccines based on the PCV2a subtype [6, 20, 21]. It was demonstrated that the current PCV2a subtype vaccines provide complete protection against PCV2b challenge [26, 28]. Moreover, Opriessnig et al. showed that the PCV1-2b vaccination significantly reduced the prevalence and amount of PCV2b viremia compared with the PCV1-2a vaccination [25]. Currently, many swine producers and veterinarians prefer to use single-dose administration because of decreased labor costs. It has been reported that one-dose and two-dose PCV2 vaccine products were equivalent in inducing protective immunity, and the major disadvantage of not giving a booster dose is the lack of generating a larger number of memory B cells resulting in a longer lag period after encountering with the antigen [22]. We have previously tested the efficacy of a two-dose inactivated PCV1-2b vaccine using PCV2b challenge [29]. In this study, we determined the efficacy of one-dose inactivated or live-attenuated (2 × 10^3.5^ or 2 × 10^4.0^ TCID_50_ dose) PCV1-2b vaccines in a wild-type PCV2b challenge model.

The optimal vaccination protocol of the inactivated PCV1-2b vaccine was determined previously [30]. Experimental pigs were vaccinated with 2 × 10^4.0^, 2 × 10^5.0^ and 2 × 10^6.0^ TCID_50_ of inactivated PCV1-2b virus. The trial test results showed that vaccination with 2 × 10^5.0^ TCID_50_ of inactivated PCV1-2b virus was more effective for PCV2 challenge, and this was confirmed by the data related to the level of antibodies against PCV2, neutralizing antibodies, viral loads in serum or lymph nodes and lesion in tissues. Corresponding to our previous study [29], all vaccines tested induced a high level of neutralizing antibody in this study which may be an important deterrent for virus replication [31].

In the current study, live-attenuated chimeric vaccine-induced clinical signs were not observed and the average daily weight gain was comparable with that of pigs in the mock group, which confirmed that the live vaccine was attenuated. All vaccinated pigs had seroconversion to PCV2 by 21 DPV and peaked at 42 DPV in group 1 (average at 1:9600), at 35 DPV in group 2 (average at 1:13120), at 35 DPV in group 3 (average at 1:15680), and at 28 DPV in group 4 (average at 1:5600) (Fig. 1). The results indicated that pigs vaccinated with the inactivated or live-attenuated vaccine developed high PCV2 antibody titers, which is consistent with a previously reported correlation between live-attenuated PCV1-2b vaccine and inactivated vaccines [32, 33]. It needs to be explained that only four pigs were present for the duration of the study in the commercial inactivated PCV2b vaccine X group, which was considered reasonable for statistical analysis and should not have affected the study outcome [32]. It is notable that the capsid protein is a major viral structural protein, and molecular epidemiology analyses have shown that the ORF2 gene is highly variable compared with ORF1 and ORF3 in the PCV2 populations [34, 35]. Therefore, a novel chimeric PCV1-2b with the capsid gene of the PCV2b/1B/Jiangsu/Jingjiang/2012/11/08 cloned into the genomic backbone of PCV1 was generated; the capsid gene shares 95.3 % nucleotide sequence identity with the previous PCV1-2b-1C strain and the efficacy of protective immunity was compared in the future.

As expected, vaccination reduced PCV2 genomic copy number in serum, SILN samples and PCV2 antigen in tissues sections compared with nonvaccinated challenged pigs. PCV2 DNA levels peaked at 14 DPC and then declined. Vaccination with the 2 × 10^4.0^ TCID_50_ dose live-attenuated PCV1-2b resulted in lower PCV2 genomic copy numbers in serum than with the 2 × 10^3.5^ TCID_50_ dose live-attenuated PCV1-2b, which directly correlated to the higher IFA antibody titers in vaccinated pigs. These data may indicate that the humoral immunity induced by the vaccine interferes with PCV2 replication [33, 36]. Furthermore, no PCV2 viremia was present in live-attenuated PCV1-2b immunized pigs at 21 DPC, suggesting that live-attenuated vaccines may induce better cellular immune response, although the exact mechanism for the decrease of PCV2 viremia in vaccinated pigs is unknown. Lymphoid tissues are the main targets of PCV2 in pigs with related diseases; PCV2 replication in lymph nodes results in degradation of lymphoid structures and impairment of the immune system [37–39]. The amount of PCV2 copy numbers in SILN is important to evaluate the vaccine efficacy. At 21 DPC, PCV2 genome was detected in high amounts in SILN of nonvaccinated challenged pigs, but low amounts in vaccinated pigs. The mean PCV2 copy numbers of positive SILN samples were 8.62 ± 0.64, 8.70 ± 0.54, 7.89 ± 0.39, and 9.08 ± 0.77 in vaccinated groups, respectively, compared with 11.5 ± 0.30 in the nonvaccinated challenged group, the difference was statistically significant (*P* < 0.05). The distribution of PCV2 antigens was described by IHC, PCV2 antigen was undetected in the 2 × 10^4.0^ TCID_50_ dose live-attenuated vaccine group and detected only in one pig in the inactivated or 2 × 10^3.5^ TCID_50_ dose live-attenuated PCV1-2b vaccine group, and the commercial inactivated PCV2b vaccine X group was positive for PCV2 antigen in lymphoid tissues. In contrast, all pigs tested were positive in the nonvaccinated challenged group. These findings are in agreement with a previous study [29] and imply that inactivated and live-attenuated PCV1-2b vaccines can significantly reduce the amount of PCV2 virus in serum or lymphoid tissues, which are characteristic factors in the pathogenesis of PCVAD in pigs [40, 41].

In this study, the observed PCV2-associated microscopic lesions were milder in the lung and lymphoid tissues in all vaccinated pigs compared with the nonvaccinated challenged pigs. In addition, the clinical signs of disease were not observed in the vaccinated pigs. We found that in nonvaccinated challenged pigs, LD and HR of follicles were associated with high PCV2 viral copy numbers and more serious histological lesions in lymph node, spleen, and tonsil which may eventually progress to clinical PMWS [37, 42]. In contrast, vaccinated pigs with high antibody titers presented mild microscopic lesions and PCV2 viremia after challenge.

## Conclusions

In summary, the data from this study demonstrate that pigs vaccinated with inactivated or live-attenuated chimeric PCV1-2b vaccines were equally protected against PCV2 challenge, as evidenced by the lack of PCV2 viremia, significant reduction in viral loads in lymphoid tissues and significantly less lymphoid lesions in vaccinated pigs. This chimeric PCV1-2b virus may be a good candidate not only as an inactivated vaccine but also as a live-attenuated vaccine against PCV2 infection.

## Materials and methods

### Cells and viruses

PCV-free PK-15 cells used in this study were purchased from the China Institute of Veterinary Drug Control and grown in Dulbecco’s modified Eagle’s medium (DMEM; Sigma), supplemented with 4 % fetal bovine serum (FBS; Gemini). The construction of the recombinant pBSK(+)-dPCV1-2b plasmid was reported previously [29]. PCV2b/1B/Jiangsu/Jingjiang/2012/11/08 isolated from a pig with clinical manifestation of PMWS was sequenced for the entire viral genome (GenBank accession no. KJ599673).

### Vaccine preparation

Chimeric PCV1-2b live viruses were generated by electroporation of PK-15 cells as previously reported [43]. Briefly, the trypsinized PK-15 cells were transferred to a sterile electroporation microcuvette containing 6 μg of plasmid DNA resuspended in 400 μL of transfection buffer. The electroporation parameters were three 400-μs pulses at 250 V. After electroporation, fresh DMEM containing 4 % FBS was added and transferred to six-well plates, which were incubated at 37 °C with 5 % CO_2_ for 48 h. Cells were then harvested.

To determine the infectivity titers of the chimeric PCV1-2b viruses, PK-15 cells were cultivated on 96-well plates, and then the cells were infected with a 10-fold serial dilutions of PCV1-2b. After 72 h of incubation, the virus titers were determined by an indirect immunofluorescence assay (IFA) [29, 43]. Briefly, the infected cells were fixed with methanol and then incubated with PCV2 antiserum (1:1000 dilution; VMRD). After washing three times with phosphate-buffered saline (PBS; 0.01 M, pH 7.2), the cells were incubated with fluorescein isothiocyanate (FITC)-labeled rabbit anti-pig IgG (Southern Biotech). Virus titers were determined using a fluorescence microscope (IX51; Olympus).

Then, the chimeric PCV1-2b viruses were diluted to 10^3.5^ TCID_50_/ml and 10^4.0^ TCID_50_/ml and were regarded as live-attenuated PCV1-2b vaccines. The inactivated chimeric PCV1-2b vaccine was developed using the following steps. Briefly, live chimeric viruses with a titer of 10^5.5^ TCID_50_/ml were incubated with formalin at a final concentration of 0.1 %, kept at 4 °C for 12 h, and spun at 120 rpm at 37 °C for 12 h. Virus inactivation was confirmed by the inoculation of the formalin-treated samples into PK-15 cells and serially passaged for two generations, and then tested by IFA. Next, the inactivated PCV1-2b viruses were mixed with ISA 206 adjuvant (Seppic, Paris, France) at a ratio of 46:54 (V/V), and the inactivated chimeric PCV1-2b viruses in the prepared vaccine reached 10^5.0^ TCID_50_/ml.

### Experimental design

The experimental protocol was previously approved by the Animal Care and Use Committee of Yangzhou University (approval ID: SYXK [Su] 2005–0005). Twenty-seven 3-week-old conventional pigs were randomly divided into six groups, including four vaccination groups and two control groups (Table 1). All pigs were negative for PCV2, porcine reproductive and respiratory syndrome (PRRSV), porcine parvovirus (PPV), porcine pseudorabies virus (PRV), and classic swine fever virus (CSFV) according to IFA and polymerase chain reaction (PCR) tests. Each group of pigs was housed separately. The pigs in groups 1 to 4 were vaccinated in the right side of the neck at 3 week old. Live-attenuated PCV1-2b was given as 2 ml × 10^3.5^ TCID_50_ and as 2 ml × 10^4.0^ TCID_50_ (groups 1 and 2), respectively; inactivated PCV1-2b vaccine was given as 2 ml × 10^5.0^ TCID_50_ (group 3). A commercial inactivated PCV2b vaccine named X, which is available on Chinese market, was given as a 2.0-ml dose as recommended by the manufacturer (group 4). Phosphate-buffered saline (PBS; 0.01 M, pH 7.2) was given as a 2.0-ml dose (groups 5 and 6). At 28 days post vaccination (DPV), pigs in all groups, except for the mock group, were challenged with 2 ml of wild-type PCV2b/1B/Jiangsu/Jingjiang/2012/11/08 at a titer of 10^4.8^ TCID_50_/ml. After challenge, all pigs were monitored daily for clinical signs of diseases. Blood samples were collected at −1, 7, 14, 21, and 28 DPV and at 7, 14, and 21 DPC until necropsy.

### Clinical evaluation

Pigs were weighed at 0 DPV and weekly thereafter until the day of necropsy. Rectal temperatures and clinical observations, including evidence of central nervous system disease, icterus, musculoskeletal disease, and changes in body condition, were recorded daily [20].

### Titer of anti-PCV2 IgG antibodies and neutralizing antibodies

The serum samples were tested for the presence of PCV2 antibodies by IFA [21]. Briefly, 200 TCID_50_ PCV2 virus was added to PK-15 cells that were grown in 96-well plates. After 72 h incubation, the infected cells were fixed with methanol for 15 min and then incubated with the serum specimen diluted in PBS at 1:10, 1:100, 1:200, 1:400, 1:800, 1:1600, 1:3200, 1:6400, 1:12,800, and 1:25,600. After incubation at 37 °C for 1 h, the cells were washed with PBS and incubated with FITC-labeled rabbit anti-pig immunoglobulin G (Southern Biotech) at 37 °C for 45 min. The antibody titer was defined as the highest dilution time of serum under a fluorescence microscope (IX51; Olympus).

The titer of neutralizing antibody (NA) against PCV2 was assessed according to the fluorescence focus neutralization (FFN) assay [29, 44, 45], with minor modifications. Briefly, the tested serum samples were inactivated at 56 °C for 30 min, serially diluted two-fold (1:2 to 1:128), mixed with 200 TCID_50_ PCV2 virus at an equal volume ratio and incubated for 1 h at 37 °C. The serum-virus mixture was added to confluent PK-15 cells growing in 96-well plates. After incubation at 37 °C for 72 h, a similar IFA protocol was performed. Serum NA titer was determined as the highest dilution at which there was 90 % or greater reduction in virus replication compared with the virus control.

### Quantification of PCV2 DNA in blood and superficial inguinal lymph nodes

All serum samples at 7, 14, and 21 DPC and superficial inguinal lymph nodes (SILN) were tested for amount of PCV2 DNA. A pair of primers (5′-TGGCCCGCAGTATTCTGATT-3′ and 5′-CAGCTGGGACAGCAGTTGAG-3′), and a probe (5′-6FAM-CCAGCAATCAGACCCCGTTGGAATG-TAMRA-3′) were designed to target a highly conserved region in PCV2 ORF1 according to a previously reported method [24]. Viral DNA was extracted from 100 μl of serum samples and 50 μg of SILN tissue; resulting DNA was suspended in 100 μl of sterile water [20]. The quantitative real-time PCR assays were performed on a Roche Real-Time PCR Detection System (LightCycler® Nano, Roche, Basel, Switzerland) using a Roche FastStart Essential DNA Probes Master Kit (Roche). Samples were considered negative when no signal was observed during the 40 amplification cycles. All reactions were performed in triplicate.

### Histopathology

All pigs underwent necropsy and were evaluated by veterinary pathologists in a blinded fashion at 21 DPC. Sections of lung (five sections), heart, tonsil, thymus, liver, spleen, small intestine, colon, kidney, and lymph nodes (tracheobronchial, mediastinal, mesenteric, subiliac, and superficial inguinal) were collected, fixed in 10 % neutral buffered formalin, and routinely processed for histological examination.

### Immunohistochemistry

Immunohistochemistry (IHC) for detection of PCV2-specific antigen was performed on selected formalin-fixed and paraffin-embedded sections of lymph nodes, tonsil, spleen, and thymus tissues using a pig polyclonal antiserum (VMRD) [46]. The amount of PCV2 antigen distributed was scored by veterinary pathologists in a blinded fashion. Scores ranged from 0 for no signal to 3 for more than 50 % with PCV2 antigen staining [47].

### Statistical analysis

Statistical analysis was performed using SPSS software (version 13.0). Viral copy loads in serum samples or SILN tissue were transformed to values of log_10_ viral copy with Graphpad Prism v5.0 software package (Graphpad Software, La Jolla, CA) and expressed as means with standard deviations. The *t* tests (and nonparametric tests) were used to draw the graphs to display PCV2 viral copy loads in serum samples or SILN tissue and the dynamics of seroconversion to PCV2-specific IFA antibody.
